# International Medical Congress

**Published:** 1873-05

**Authors:** 


### INTERNATIONAL MEDICAL CONGRESS.

We learn from the pages of the *Berlin Clinical Weekly* that the third session of the International Medical Congress will be held at Vienna, Austria, during the time of the World's Fair, from the 2d to the 10th of September next.

As members of the Congress are considered: 1. The members of all committees entrusted with the preliminary labors. 2. Delegates of governments and of all scientific corporations (universities, academies, medical associations, hospitals.) 3. All physicians and naturalists generally, who report their names before the day of meeting, to the President. No assessments or taxation for membership will take place. The sessions are public, and every member has a right to participate in the discussions and in the voting. The programme of the labors of the session consists: 1. In the discussion of the subjects chosen by the Executive Committee, on the basis of reports and propositions prepared at the instance of the Committee; and, 2. Discussion of questions which have been announced to the President up to May 1, and have been embraced in the order of business of a certain day.

Rokitansky is President of the Executive Committee, and thereby President of the Congress. The subjects, which so far have been chosen for discussion, are the following: 1. The question of vaccination, by Hebra. 2. The question of quarantine in relation to cholera, by Oser, and by Drasche. 3. The question of the sanitary improvement of cities, by Schauenstein. 4. The question of prostitution, by Sigmund. 5. Propositions for the establishment of an international pharmacopœia, by Bernatzik. 6. Propositions for the establishment of the greatest possible uniformity in the study of medicine in all countries, and a corresponding legal privilege of regularly educated physicians to practice medicine in all countries, by Schneller and Benedikt.

The language of the third session of the International Medical Congress will be German, but other languages are admissible during the discussion. The communications of the President are made in German; but French, English, and Italian translations are furnished. The same rule has been adopted for the publication of the transactions.

